# Metabolic Hepatic Disorders Caused by Ciguatoxins in Goldfish (*Carassius auratus*)

**DOI:** 10.3390/ani12243500

**Published:** 2022-12-11

**Authors:** María José Ramos-Sosa, Víctor Hernández López, Andres Sanchez-Henao, Natalia García-Álvarez, Ana R. Díaz-Marrero, Inmaculada Rosario, Fernando Real, José J. Fernández

**Affiliations:** 1 Division of Fish Health and Pathology, University Institute of Animal Health and Food Safety (IUSA), University of Las Palmas de Gran Canaria, 35416 Arucas, Gran Canaria, Spain; 2 Instituto Universitario de Bio-Orgánica Antonio González (IUBO AG), Universidad de La Laguna (ULL), Avenida Astrofísico Francisco Sánchez 2, 38206 La Laguna, Tenerife, Spain; 3 Departamento de Química Orgánica, Universidad de La Laguna (ULL), Avenida Astrofísico Francisco Sánchez s/n, 38206 La Laguna, Tenerife, Spain; 4 Instituto de Productos Naturales y Agrobiología (IPNA), Consejo Superior de Investigaciones Científicas (CSIC), Avenida Astrofísico Francisco Sánchez 3, 38206 La Laguna, Tenerife, Spain

**Keywords:** ciguatoxins, metabolomics analysis, ciguatera poisoning, nuclear magnetic resonance, goldfish, liver, hepatosomatic index, ammonia

## Abstract

**Simple Summary:**

Ciguatoxins (CTXs) are potent neurological marine toxins able to cause ciguatera poisoning (CP) in humans via the food web. Although considered one of the most important food-borne diseases in the world, little is known about how CTXs are metabolized in fish, and the possible alteration of hepatic metabolomic linked to these toxins. To clarify this aspect, goldfish (*Carassius auratus*) were fed with C-CTX1 enriched flesh, and their livers were analyzed by nuclear magnetic resonance (NMR). Altered concentrations of glucose, glycogen, taurine, alanine, and lactate were observed. Furthermore, an increase in liver size was noted in animals fed a toxic diet. In addition, increased ammonia concentrations in the aquarium with toxic feed were detected. These results suggest that C-CTX1 ingestion leads to changes in liver metabolomic, which are related to the glucose-alanine cycle. This is the first evidence of CTXs-mediated alterations in liver metabolomic, and a step to further elucidate the metabolism of these toxins within fish.

**Abstract:**

Ciguatera poisoning (CP) is a foodborne disease known for centuries; however, little research has been conducted on the effects of ciguatoxins (CTXs) on fish metabolism. The main objective of this study was to assess different hepatic compounds observed in goldfish (*Carassius auratus*) fed C-CTX1 using nuclear magnetic resonance (NMR)-based metabolomics. Thirteen goldfish were treated with C-CTX1-enriched flesh and sampled on days 1, 8, 15, 29, 36, and 43. On day 43, two individuals, referred to as ‘Detox’, were isolated until days 102 and 121 to evaluate the possible recovery after returning to a commercial feed. At each sampling, hepatic tissue was weighed to calculate the hepatosomatic index (HSI) and analyzed for the metabolomics study; animals fed toxic flesh showed a higher HSI, even greater in the ‘Detox’ individuals. Furthermore, altered concentrations of alanine, lactate, taurine, glucose, and glycogen were observed in animals with the toxic diet. These disturbances could be related to an increase in ammonium ion (NH_4_^+^) production. An increase in ammonia (NH_3_) concentration in water was observed in the aquarium where the fish ingested toxic meat compared to the non-toxic aquarium. All these changes may be rationalized by the relationship between CTXs and the glucose–alanine cycle.

## 1. Introduction

Ciguatoxins (CTXs) are polyether compounds responsible for ciguatera poisoning (CP), which is one of the most important fish and shellfish-borne diseases worldwide [1]. Three families of CTXs have commonly been classified according to their geographical location, i.e., Pacific (P-CTXs), Caribbean (C-CTXs), and Indian Ocean (I-CTXs) as reviewed [2]. The presence of C-CTX1 has been reported in species from the Canary Islands and the Selvagens Islands [3,4,5], including species associated with human poisonings in these areas [6].

Currently, the metabolization of the CTX inside the organisms is not clear. The liver is considered the primary organ for the detoxification and storage of lipophilic compounds [7] and the CTX content in fish hepatic tissue is typically much higher than that in muscle [8]. In addition, the hepatosomatic index (HSI) provides information about the energy status of the liver and it is used to assess the health of fish individuals. Few studies utilizing genomics and proteomics investigations have been conducted on the impacts of CTX in fish to assess potential biomarkers and resistance mechanisms, mainly involving the CTX1B [7,9]. Regarding C-CTX, less research has been performed except for a recently published study by Gwinn et al. [10], where the first evidence of CTX glucuronidation in fish was proposed, suggesting that it could be a prevalent detoxification mechanism.

Metabolomics allows the detection of variations in metabolites among tissue types throughout an organism. This strategy has made it possible to study the disturbances caused by environmental changes and toxic exposures. In the last few years, many studies have been conducted to observe the alterations caused by xenobiotics in organisms, showing its great value for toxicological studies, as reviewed by Bonvallot et al. [11], and references therein. In the case of CTXs, there exist studies on toxin accumulation and depuration in some fish models [12,13]; however, there is no research on how these toxins affect the correct functioning of organs. Therefore, nuclear magnetic resonance (NMR) spectroscopy has become one of the most important analytical techniques for metabolomics studies because it is a nondestructive procedure, being highly automatable and reproducible, allowing the rapid detection and characterization of a wide variety of compounds [14].

In this study, the goldfish (*Carassius auratus*), a member of the *Cyprinidae* family which includes other carps such as the common carp (*Cyprinus carpio*) and Crucian carps (genus *Carassius*), has been used as model fish species. It is an omnivorous fish with a high resistance to temperature fluctuations, low dissolved oxygen levels, and a high tolerance range for pH. These characteristics, among others, make it a widely used species as a model for the scientific community, reviewed by Blanco et al. [15]. Furthermore, the goldfish is considered a highly ammonia-tolerant breed [16]. Several studies have been performed on *C. auratus* to assess the metabolomics alterations caused by pollutants [17,18,19]. Moreover, in a recent study published by Sanchez-Henao et al. [20], it was observed that goldfish can accumulate CTX in muscle.

In the experimental survey, the control of the ammonia parameter is critical for the survival of fish. In aqueous solutions, ammonia is present as unionized ammonia (NH_3_) and ammonium ion (NH_4_^+^). Both forms exist in equilibrium (NH_3_ + H_3_O^+^ ↔ NH_4_^+^ + H_2_O), and normally the general term ammonia will refer to both forms. In freshwater fish species, NH_3_ diffuses easily through biological membranes, while these membranes are usually less permeable to ionic compounds; therefore, toxic effects are related to the accumulation of NH_3_ in the environment. However, within the organism, it is the high levels of NH_4_^+^ that present the strongest hazard [21]. Ammonia values are increased after feeding, with the liver being its main producer due to the metabolism of amino acids, and it is excreted mainly by the gills, reviewed by Bucking [22].

To the best of our knowledge, no studies of CTX effects on fish liver metabolomics have been published to date. For that purpose, this study aimed to evaluate the effect of C-CTX1 on the liver metabolism of the goldfish using HSI, ammonia release, and an NMR-based metabolomics approach. This strategy provides information on the metabolic effects of C-CTX1.

## 2. Materials and Methods

### 2.1. Fish, Experimental Design, and Animal Welfare

Two experimental trials were carried out with goldfish (*C. auratus*). The first, described in this section, was to evaluate the liver metabolomics changes of fish fed with C-CTX1-contaminated flesh. The second, described in Section 2.5, was performed in parallel, to assess ammonia levels in the water. This assay was conducted separately to avoid disturbing the fish of the first assay.

For the first experiment, adult goldfish individuals (*n* = 26) were obtained from a local commercial fish distributor from Las Palmas de Gran Canaria, Spain. Detailed procedure and flesh CTX-like toxicity results of this research have been previously published [20].

Briefly, after a period of acclimation, fish were arbitrarily separated into two groups: the experimental (*n* = 13) and control (*n* = 13) groups. On the first day of the experiment, three individuals from the control group were sacrificed to determine the normal liver metabolomics profile of goldfish fed with commercial feed. After that, the experimental group was fed flesh from an amberjack naturally contaminated with C-CTX1, as confirmed by liquid-chromatography–mass-spectrometry (LC-MS/MS) (0.270 ng·g^−1^) by the University of Vigo; whereas, the control group was fed with non-toxic amberjack flesh showing a CTX level below the limit of detection (LOD) observed by a neuroblastoma (Neuro-2a)-cell-based assay (CBA) [20]. To ensure the total consumption of the experimental diet, the raw flesh was homogenized in agarose gel. Experimental fish received 0.014 ng CTX1B equivalents (Eq.)·(g of fish weight)^−1^ in their food every day; each feeding was supervised to verify the correct ingestion of the corresponding daily dose.

Fish were sampled after 1, 8, 15, 29, 36, and 43 days of daily toxic feeding. Control and experimental fish were analyzed in each sampling and livers from both groups were collected for metabolomics study. Each sampling was performed 24 h post-feeding and before any new exposure. After sacrifice, fish were immediately dissected, and the liver was frozen at −80 °C for the metabolomics determination (four liver samples for each experimental individual). All the measures of body weight (nearest g) and length (nearest cm) were collected before freezing.

On day 43 of the experiment, three fish showed severe symptoms while feeding and one of them was sampled and analyzed. The other two fish were isolated to assess a possible recovery after changing their diet back to commercial feed, one up to day 102 and the other one until day 121 of the experiment. These animals were identified as ‘Detox’.

The experimental protocol was approved by the Committee for Animal Welfare of the University of Las Palmas de Gran Canaria and by the Department of Agriculture, Livestock, Fisheries and Water of the Canary Islands Government (code no. OEBA-ULPGC 28/2018).

### 2.2. Hepatosomatic Index (HSI)

Immediately after sampling, the liver was weighed, to the nearest 0.001 g, to determine the HSI to obtain information on liver condition and energy content. HSI was calculated as:

$$\mathrm{HSI}=\frac{\mathrm{Liver}\;\mathrm{weight}\;\left(g\right)}{\mathrm{Body}\;\mathrm{weight}\;\left(g\right)}\times 100$$

### 2.3. Sample Preparation and NMR Spectroscopy

Liver tissue samples of *C. auratus* (four samples per individual, 43.3 ± 2.9 mg) from the experimental (*n* = 52) and control (*n* = 52) were extracted by addition of 1.5 mL acetonitrile/water (1:1). The tissue was homogenized using a SONICS ultrasonic processor equipped with a 3 mm ultrasound probe in 30 sec pulse periods up to 8 min followed by cooling on dry ice for 15 min. After centrifugation for 15 min at 15,000× *g* at 4 °C, 1.0 mL of the supernatants was transferred to empty vials and concentrated in a Thermo Scientific Savant SpeedVac Vacuum Concentrator (Thermo Fisher Scientific Inc., Oxford, UK) at room temperature. The dry samples were reconstituted in phosphate buffer (600 μL of a 0.2 M solution) containing D_2_O, NaN_3_ (3 mM), and TSP (1 mM). The ^1^H NMR and HSQC spectral data were acquired at 298 K on spectrometers Bruker Avance-500 (Bruker Biospin, Falländen, Switzerland) and Avance-600 equipped with a TCI cryoprobe, respectively. For each experiment, 60 transients were collected into 64 k data points using a spectral width of 7507.5 Hz (acquisition time of 2.18 s) and a relaxation delay of 1 s. All spectra were processed with the software MestReNova (V11.0, Mestrelab Research S.L.). Afterward, all spectra were phase- and baseline-corrected. Finally, the chemical shifts were referenced to the TSP signal at 0.0 ppm. Each spectrum was normalized using the TSP signal as a standard for comparative analyses.

### 2.4. Metabolomics Correlation and Pathway Analysis

Metabolic content signal analysis of 2D HSQC was performed to determine the toxic effects of the CTXs-enriched diet on the liver metabolic pathways of the treatment and control groups. The ^1^H and ^13^C NMR chemical shifts obtained from the HSQC experiments of the selected representative samples (toxic and non-toxic) were analyzed using the online COLMAR ^13^C-^1^H HSQC metabolomics database (http://spin.ccic.ohio-state.edu/index.php/hsqc/index (last accessed on 28 September 2022); Campus Chemical Instrument Center of The Ohio State University) [23] to identify metabolites present in the samples.

### 2.5. Determination of the Ammonia Level Variations

Two aquaria with similar fish biomass (63.22 g and 62.56 g, respectively), amount of water, filters, types, and amount of biofilter materials (317.45 g and 318.34 g, respectively) were used to determine the possible ammonia (NH_3_) concentration alterations. Fish (*n* = 4) from one aquarium received toxic raw amberjack flesh, whereas the other one (*n* = 4) received non-toxic raw amberjack flesh. The day before the beginning of the experiment, biofilter materials were collected, mixed, and divided into the two filters, to guarantee the same conditions in both aquaria.

Ammonia levels were recorded 14 days out of the 16 days of the experiment. Measurements were conducted after 24 h post-feeding. On days 5 and 12, the individuals were not fed, receiving food 6 days a week to study the possible variation in the ammonia concentration. Ammonia levels were determined with a freshwater ammonia colorimeter—checker^®^ HC (Hanna instruments Inc., Woonsocket, RI, USA). Two testers were used: freshwater low-range ammonia colorimeter—checker^®^ HC (HI700; range: 0.00 to 3.00 ppm NH_3_-N; resolution: 0.01 ppm; accuracy at 25 °C: ±0.05 ppm ±5% of reading), and medium-range ammonia colorimeter—checker^®^ HC (HI715; range: 0.00 to 9.99 ppm NH_3_-N; resolution: 0.01 ppm; accuracy at 25 °C: ±0.05 ppm ±5% of reading), depending on the ammonia levels. The method followed by these checkers is an adaptation of the *ASTM Manual of Water and Environmental Technology, D1426, Nessler Method*.

The ammonia checker used displays the amount (mg/L, ppm) of ammonia nitrogen (NH_3–_N) present in the water. According to the manufacturer, to convert the results in ammonia levels (ppm), the following formula was used:

$$\mathrm{Ammonia}\;\left(\mathrm{ppm}\right)=\mathrm{Ammonia}\;\mathrm{nitrogen}\;\left({\mathrm{NH}}_{3}-N\right)\times 1.214$$

On day 9, the water was renewed due to the high ammonia values detected to ensure the welfare of the animals. To ensure equal conditions, the same amount was replaced in both aquaria (75% of the total). Besides NH_3_, other water parameters were checked to guarantee good aquarium conditions, as shown in Tables S3 and S4.

### 2.6. Statistical Analysis

To evaluate the HSI values, data analysis was conducted using PASW Statistics software 18.0 for Windows (SPSS Inc., Chicago, IL, USA).

The non-parametric test Kruskal–Wallis was used to compare the HSI values according to the food received, due to the small number of individuals analyzed. As usual, a *p*-value ≤ 0.05 was considered statistically significant.

## 3. Results

### 3.1. Hepatosomatic Index (HSI) Variations

**Figure 1:**
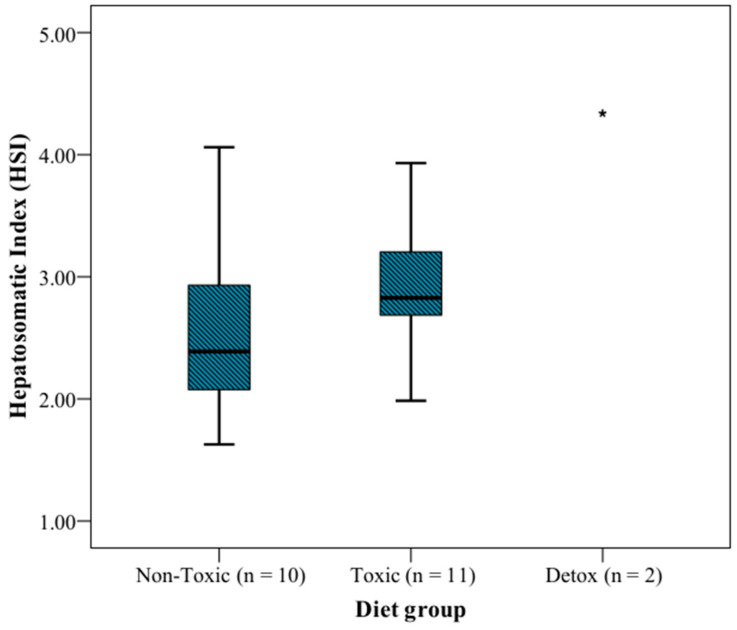
Hepatosomatic index (HSI) of the different diet groups. The plot represents the interquartile range (Q3–Q1), and the dark line draws the median values. * Indicates the mean value of the detox group.

The HSI was determined in each individual analyzed in this study (Table S1). It was observed that the group fed toxic flesh (*n* = 11) showed a higher HSI than the group fed non-toxic flesh (*n* = 10) (2.827 and 2.388 as median, respectively) (Table 1 and Figure 1). The two specimens separated at day 43 after ingesting toxic food and then fed commercial food for possible recovery, showed the highest HSI (3.907 and 4.755, respectively).

Figure 1 depicts the HSI variations depending on the diet groups studied. Although a clear disparity with the detox group is apparent, the difference is not significant between the non-toxic and toxic groups ($x^{2}\left(1\right)=1.269;p=0.260$).

### 3.2. Metabolic Profiles of Liver Samples of Carassius auratus

**Figure 2:**
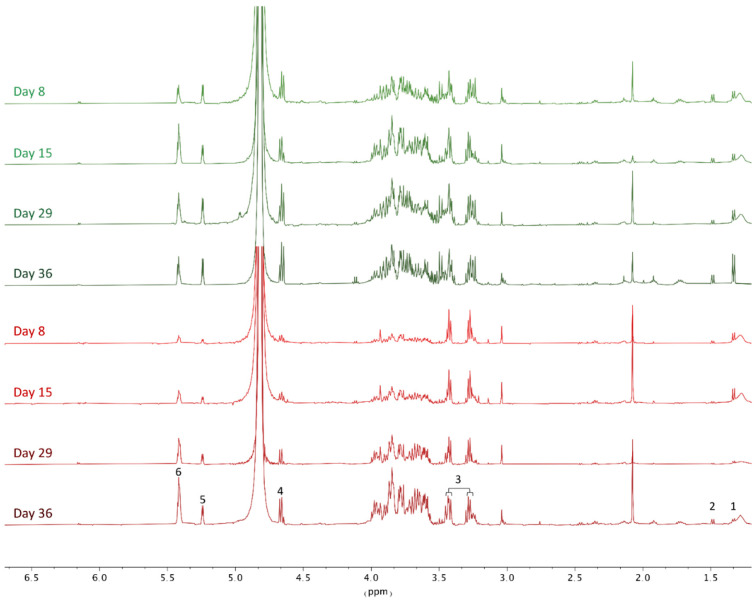
Effect of diet on the metabolic profile of liver tissues. Representative ^1^H NMR spectra (500 MHz, 298 K) of the liver samples of *Carassius auratus* fed with CTX-toxic (red) and non-toxic (green) fish diet. Effect of time of treatment after 36 days. Key metabolites: (1) lactate, (2) alanine, (3) taurine, (4) α-glucose, (5) β-glucose, (6) glycogen.

All liver tissue samples of *C. auratus* were extracted and reconstituted in phosphate buffer, containing D_2_O, NaN_3_, and TSA as internal standards for NMR analysis, as described in the experimental section. Firstly, 2D HSQC experiments were performed in both the control and treatment groups (Figures S1 and S2) to identify key metabolites present in the samples. Using the online COLMAR ^13^C-^1^H HSQC metabolomics database [23], the analysis of the ^1^H and ^13^C fingerprints of the whole metabolic content revealed by the HSQC experiments allowed us to identify the presence, characteristic signals, and their chemical shifts of metabolites such as taurine, lactate, alanine, α- and β-glucose, and glycogen with a matching ratio of 1. This ratio corresponds with the ratio between the number of matched peaks in the experimental HSQC spectrum to the number of peaks of the metabolite in the database, in which 1 corresponds to a 100% match. Once identified, the metabolic imbalance of those selected metabolites was estimated by comparison of the intensities of the signals in ^1^H NMR experiments of each metabolite, using TSP as an internal standard. Hence, the ^1^H NMR spectra of all processed liver tissue samples were normalized to the internal standard and grouped for qualitative comparative analysis. The observed metabolic effects of the CTX-enriched toxic diet on the liver of *C. auratus* at days 8, 15, 29, and 36 are shown in Figure 2 compared to untreated control specimens fed a non-toxic fish diet (see also Figures S3 and S4). The analysis reveals that, after 8 days, CTX-treated fish showed an increase of 13% in taurine concentration which remained at high levels until the end of the experiment compared to control samples. Additionally, while control liver samples show stable concentrations of lactate and alanine, a progressive decrease of the presence of both metabolites is observed in treated samples, particularly on day 29, with more than 80% reduction. Additionally, remarkable alterations were also observed concerning glucose metabolism, with a progressive accumulation of glucose/glycogen detected along the experiment in CTX-treated fish (Figure 2, Figures S3 and S4), showing an increase of 80% from day 8 to day 36.

### 3.3. Ammonia Levels

**Figure 3:**
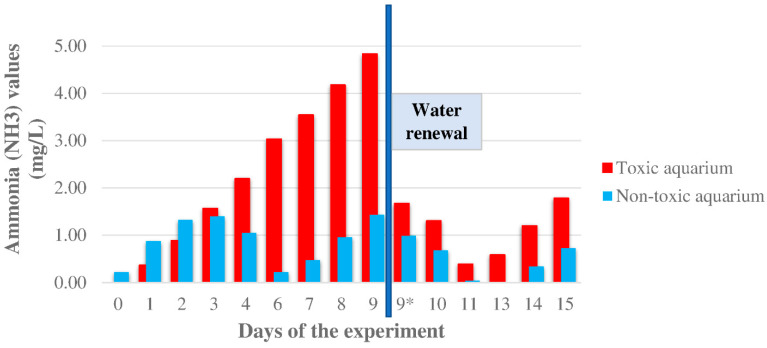
Ammonia (NH3) values (ppm) were obtained from the two aquaria during the experiment. A water renewal was performed on day 9 (blue line). Fish were not fed on days 5 and 12 of the experiment. * The ammonia was measured just after the water change.

The ammonia levels are shown in Figure 3 and Table S2. Before starting the experiment, NH_3_ values were measured in both aquaria, and the non-toxic aquarium showed a higher value (0.219 ± 0.05 ppm) than the toxic one (0.000 ± 0.05 ppm). The non-toxic aquarium kept a higher value for the first two days of the experimental diet, but after the third ingestion of toxic food, the levels of the toxic aquarium were greater than the non-toxic one (1.566 ± 0.05 and 1.396 ± 0.05 ppm, respectively). The differences between the two aquaria increased gradually, with a maximum of 3.399 on day 9. That day, due to the high ammonia levels, a water change was done in both aquaria. In the first days after the renewal of water, the ammonia levels decreased gradually. Three days later, the ammonia levels in the toxic aquarium increased again, whereas in the non-toxic aquarium, the concentration reached 0.000 ± 0.05 ppm. The ammonia levels continued increasing until the end of the experiment.

## 4. Discussion

**Figure 4:**
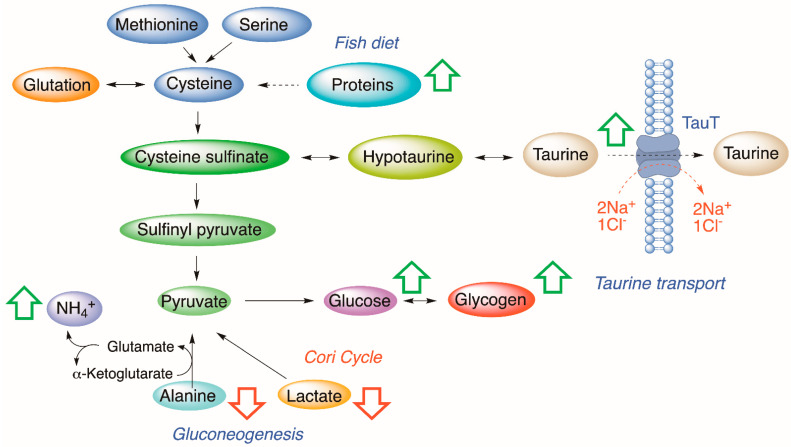
Metabolic alterations observed in the liver samples of *Carassius auratus* fed with a CTX-toxic diet compared to control samples. Green arrows represent increased metabolite concentration. Red arrows represent decreased metabolite concentration.

The experimental results demonstrate that the presence of CTXs in fish diet causes a sudden increment of taurine in the fish liver that remains at stable levels along the feeding experiment. In general terms, the concentration of taurine is determined by parameters such as biosynthesis [24], taurine transporter (TauT transporter) activity [25,26], volume sensitive flux pathways [27], liver diseases [28], as well as high-fat diet (HFD) [29], high-arginine diet [30], and high/low-protein diet [31,32,33]. Thus, at the organism level, taurine homeostasis is controlled by multiple regulatory factors. Among them, the most significant is the delivery of taurine from the extracellular to the intracellular environment, mediated by the TauT transporter. TauT transporter belongs to the group of Na^+^ Cl^−^-dependent transporters [34]. It regulates taurine movement based on the ionic environment, electrochemical charge, pH, and temperature [35]. In particular, one to three Na^+^ ions are required to elicit taurine transport [36]. The importance of sodium is confirmed through the fact that any reduction in the sodium gradient can disable further binding of taurine to the TauT transporter [37]. Fish-based diets contain high levels of proteins; however, we observed that increased levels of taurine were only detected in specimens fed with C-CTX1-toxic fish meat. The presence of CTXs in the diet clearly affects one or more of these physiological parameters which may explain the increased concentration of taurine noted in treated samples. CTXs are known to block the sodium channels causing accumulation of intracellular Na^+^ in neuronal cells [38]; hence, similarly, this might be one of the reasons for the increment of taurine observed in liver cells (Figure 4).

On the other hand, nutritional studies with marine fish species have revealed that diets supplemented with taurine restored the activity of amino acid catabolic and gluconeogenic enzymes and hexokinase to control levels [39,40]. Despite the fact that the taurine effect on glucose homeostasis has been little studied, it may be of special interest, particularly for carnivorous fish species. Actually, carnivorous fish are traditionally considered to be glucose intolerant due to prolonged hyperglycemia observed after a high glucose intake [39,41,42,43].

The efficiency of carbohydrate metabolism in fish mainly depends on the enzyme activity, insulin receptors, rate of glucose transport, and regulation efficiency of hepatic glucose utilization [44]. Dietary taurine supplementation increased the activity of intestinal amylase, glucose phosphorylation, and the activity of hepatic glucose-6-phosphate dehydrogenase (G6PD) in different fish species [45,46,47], while decreasing the catabolic enzyme activity of glucogenesis [39]. Taurine has blood-glucose-reducing properties via interactions with the insulin receptors. It was found that dietary taurine supplementation decreased the plasma glucose levels in white seabream (*Diplodus sargus*) [48]. Moreover, dietary taurine supplementation increased the glucose tolerance ability of turbot (*Scophthalmus maxima*) [45]. Under the effects of a CTX-toxic diet, we detected an increase in hepatic glucose.

**Figure 5:**
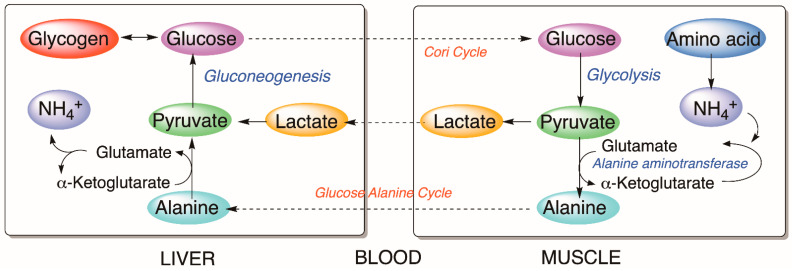
Cori and glucose–alanine cycles.

Most of the observed metabolic alterations seem to be related to the glucose–alanine cycle (Figure 5), which correlates with the metabolism of glucose in muscle and liver in mammals. Alanine is another source of gluconeogenesis in the liver and is produced from the transamination process in non-hepatic tissues. During transamination, one α-amino acid donates the amino group and generates one α-keto acid. Through the glucose–alanine cycle, the non-hepatic tissues transfer the amino moiety of the catabolized amino acids to the liver to be excreted as urea; therefore, nitrogen is eliminated from muscles while replenishing its energy supply [49]. Assuming similar metabolic pathways in fish liver, the detected effects suggest an activation of gluconeogenesis that leads to the reduction of lactate and alanine in hepatic cells; therefore, an increment of glucose, glycogen, and NH_4_^+^, as NH_3_ in water, is detected in treated fish.

**Figure 6:**
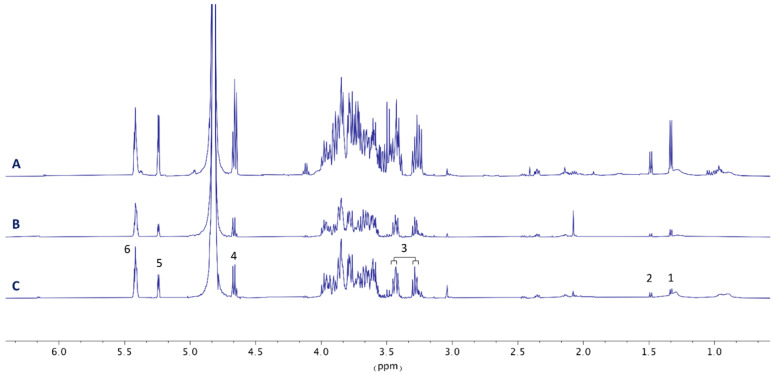
Analysis of liver detoxification. Comparison of ^1^H NMR spectra (recorded at 500 MHz, 298 K) of the liver samples of *Carassius auratus.* (A) Control fish fed with a commercial diet. (B) Fish fed with 43 days of toxic diet with severe symptoms. (C) Fish fed with commercial diet for 60 days after 43 days of toxic diet and severe symptoms. Key metabolites: (1) lactate, (2) alanine, (3) taurine, (4) α-glucose, (5) β-glucose, (6) glycogen.

Specimens of *C. auratus* showing CTXs toxic syndrome on day 43 were fed with a commercial diet for at least 60 additional days. A comparison of ^1^H NMR liver metabolic profiles revealed that the toxic effects are sustained with time and that recovery of initial metabolic levels is not produced in the period analyzed (Figure 6). However, an increment of the HSI was observed in these animals. Nonetheless, an extensive experiment will be required to support these preliminary findings.

The accumulation of CTXs in the diet of fish involves an important metabolic alteration in the liver. Indeed, previous results published by Sanchez-Henao et al. [20] showed that the greatest alterations observed from day 29—increased glucose and glycogen levels, and decreased alanine and lactate concentrations—were related to the highest CTX-like toxicity found in the trial, and the moment when the concentration seemed to stabilize until the end of the experiment. Firstly, the effects observed in the liver appear to be related to a modification of taurine levels with all the changes that it triggers. Additionally, a hyperglycemia-like response is produced with high intrahepatic glucose and glycogen levels, and low lactate and alanine levels. As a consequence of the activation of gluconeogenesis, an increase in NH_4_^+^ concentration is observed. Therefore, an increment of NH_3_ levels was produced in the aquarium with fish fed with toxic flesh, shown in Figure 3 and Table S2. It is noted that on day 6 of the experiment, NH_3_ concentrations decreased in the non-toxic aquarium. This could be explained by the absence of feeding on the previous day, and therefore the reduction in ammonia excretion. In the toxic aquarium, no decline was observed on that day, probably because the animals continued to excrete NH_3_ because of CTXs detoxification. The differences between the two aquaria increased gradually, with a maximum of 3.4 ppm after 9 days in which a water renewal was performed (Figure 3). In the first days after the water renewal, a reduction in NH_3_ levels was appreciated. This reduction is produced by a more efficient transformation into other nitrogen compounds by the bacteria of the aquaria, considering that these bacteria were under higher ammonia concentrations the previous days. Nonetheless, it can be observed that the tendency of NH_3_ increment continues in the water renewal cycles.

Increased levels of glucose and glycogen in the hepatic tissue could be the reason why animals fed toxic flesh showed a higher HSI than the non-toxic group (Figure 1), since this index is linked with the liver energy status, mainly related to glycogen reserves [50]. Moreover, the two individuals isolated to determine the recovery after return to the commercial feed, showed higher HSI than the individuals from the toxic group. This may be because, although HSI is considered an indicator of the general condition of fish, stressful situations, exposure to pesticides, and intoxications could increase the liver size [51,52]. This alteration has been associated with the isolation of pollutants in liver lipid droplets, degenerative changes in liver tissue, increased detoxification capacity, and glucose-associated modifications in liver metabolism [52,53,54]. In a study published by Gwinn et al. [10], it was noted that liver glucuronidation could be an important way to depurate C-CTX1. Moreover, Diniz et al. [55] detected an increase in HSI with the increment of pollutants concentration in goldfish. This increase could be explained by the increment of cytochrome P4501A (CYP1A) synthesis related to the metabolism of xenobiotics. Thus, the highest HSI value found in the detox fish of the present research may be related to the increased activity of the liver to detoxify the CTXs.

## 5. Conclusions

*Carassius auratus* has been described as an excellent model species for studies on CTXs accumulation in tissues and the analysis of their toxic effects. Due to this fact, in this work we have been able to analyze the effects of these toxins in the liver, which is considered the target organ for detoxification. Thus, from the metabolic point of view, CTXs cause an important disruption in concentrations of taurine, alanine, and lactate, as well as glucose and glycogen in the liver. These alterations affected the hepatic size, with an increment of the HSI in animals that ingested toxic flesh. In addition, a relevant increment of NH_3_ levels was detected in aquaria with fish fed with CTXs. Although further research should be done, all these parameters can be rationalized through the relationship of CTXs to the glucose–alanine cycle.

## Figures and Tables

**Table 1:** Hepatosomatic index (HSI) details are represented as mean ± standard deviation (SD), median, minimum, and maximum values. “*n*” represents the number of individuals in each group.

Hepatosomatic Index (HSI)			
Diet group	Non-toxic *n* = 10	Toxic *n* = 11	Detox *n* = 2
Mean ± SD	2.605 ± 0.789	2.899 ± 0.572	4.331 ± 0.599
Median	2.388	2.827	4.331
Minimum	1.627	1.985	3.907
Maximum	4.061	3.931	4.755

## Data Availability

The data presented in this study are available on request from the corresponding authors.

## Supplementary Materials

The following supporting information can be downloaded at: https://www.mdpi.com/article/10.3390/ani12243500/s1, Table S1: Goldfish data and experimental conditions of each individual analyzed in this study. Body weight and liver weight are expressed in grams (g); Figure S1: HSQC experiment of control sample recorded at 600 MHz (298 K). The ^1^H and ^13^C cross peaks (ppm) of main signals are indicated; Figure S2: HSQC experiment of toxic sample after 36 days of treatment recorded at 600 MHz (298 K). The ^1^H and ^13^C cross peaks (ppm) of main signals are indicated; Figure S3: Effect of toxic diet on metabolic profile of liver tissues. Representative normalized ^1^H NMR spectra (500 MHz, 298 K) of the liver samples of *Carassius auratus* fed with CTX-toxic fish diet. Effect of time of treatment after 8 (green), 15 (ochre), 29 (orange), and 36 (purple) days. Key metabolites: (3) taurine, (4) α-glucose, (5) β-glucose, (6) glycogen; Figure S4: Effect of control diet on metabolic profile of liver tissues. Representative normalized ^1^H NMR spectra (500 MHz, 298 K) of the liver samples of *Carassius auratus* fed with non-toxic fish diet. Effect of time after 8 (purple), 15 (blue), 29 (cyan), and 36 (green) days; Table S2: Ammonia (NH_3_) values (ppm) detected in the aquaria with a precision of ± 0.05 ppm at 25 °C. Measurements were done 24 h after the last ingestion, except for the days 0, 6, and 13. Fish were not fed on days 5 and 12 of the experiment. A change of water was performed on day 9; Table S3: Water parameters (ammonia (NH_3_) values (ppm), oxygen level (ppm), dissolved oxygen saturation (%), temperature (°C), nitrite (NO_2_), nitrate (NO_3_) (ppm), and pH) detected in the CTX-feed aquaria. Measurements were done 24 h after the last ingestion, except for the days 0, 6, and 13. Fish were not fed on days 5 and 12 of the experiment. A change of water was performed on day 9; Table S4: Water parameters (ammonia (NH_3_) values (ppm), oxygen level (ppm), dissolved oxygen saturation (%), temperature (°C), nitrite (NO_2_), nitrate (NO_3_) (ppm), and pH) detected in the non-toxic-feed aquaria. Measurements were done 24 h after the last ingestion, except for the days 0, 6 and 13. Fish were not fed on days 5 and 12 of the experiment. A change of water was performed on day 9.
