# Supplementary material for: Metabolic Hepatic Disorders Caused by Ciguatoxins in Goldfish (Carassius auratus)
Source: Animals (Basel). 2022 Dec 11;12(24):3500. doi: 10.3390/ani12243500 (PMC9774503; doi:10.3390/ani12243500)
Supplement: Supplementary file 1 [file animals-12-03500-s001.zip › animals-1970877-supplementary.pdf]

Supplementary Material

Metabolic Hepatic Disorders Caused by Ciguatoxins in the Goldfish (*Carassius auratus)*

María José Ramos-Sosa[1*], Víctor Hernández López[2,3], Andres Sanchez-Henao[1], Natalia García-Álvarez[1], Ana R. Díaz-Marrero[2,4], Inmaculada Rosario[1], Fernando Real[1] and José J. Fernández[2,3]

[1] Division of Fish Health and Pathology, University Institute of Animal Health and Food Safety (IUSA), University of Las Palmas de Gran Canaria, 35416 Arucas, Gran Canaria, Spain

[2] Instituto Universitario de Bio-Orgánica Antonio González (IUBO AG), Universidad de La Laguna (ULL), Avenida Astrofísico Francisco Sánchez 2, 38206 La Laguna, Tenerife, Spain

[3] Departamento de Química Orgánica, Universidad de La Laguna (ULL), Avenida Astrofísico Francisco Sánchez s/n, 38206 La Laguna, Tenerife, Spain

[4] Instituto de Productos Naturales y Agrobiología (IPNA), Consejo Superior de Investigaciones Científicas (CSIC), Avenida Astrofísico Francisco Sánchez 3, 38206 La Laguna, Tenerife, Spain

Table S1. Goldfish data and experimental conditions of each individual analyzed in this study. Body weight and liver weight are expressed in grams (g).

Fish number	Sample day	Diet	Body weight (g)	Liver weight (g)	Hepatosomatic Index (HSI)
1	0	Commercial	46.20	0.9222	2.00
2	0	Commercial	42.04	0.7562	1.80
3	0	Commercial	47.15	1.1695	2.48
4	1	Toxic	53.66	1.5172	2.83
5	1	Toxic	46.92	0.9313	1.98
6	1	Non-toxic	39.18	0.9025	2.30
7	1	Non-toxic	40.15	0.9928	2.47
8	8	Toxic	46.00	1.8084	3.93
9	8	Toxic	43.70	1.2129	2.78
10	8	Non-toxic	42.52	1.1281	2.65
11	8	Non-toxic	32.17	0.6678	2.08
12	15	Toxic	59.38	1.6069	2.71
13	15	Toxic	55.50	1.1232	2.02
14	15	Non-toxic	49.34	1.0074	2.04
15	15	Non-toxic	36.23	1.0619	2.93
16	29	Toxic	42.75	1.3741	3.21
17	29	Toxic	63.30	2.1385	3.38
18	29	Non-toxic	45.84	1.8617	4.06
19	29	Non-toxic	47.12	0.9804	2.08
20	36	Toxic	81.21	2.5854	3.18
21	36	Toxic	41.22	1.0991	2.67
22	36	Non-toxic	35.85	0.5834	1.63
23	43	Toxic	38.31	1.2236	3.19
24	43	Non-toxic	51.65	1.9650	3.80
25	102	Detox	45.62	1.7824	3.91
26	121	Detox	66.93	3.1823	4.75

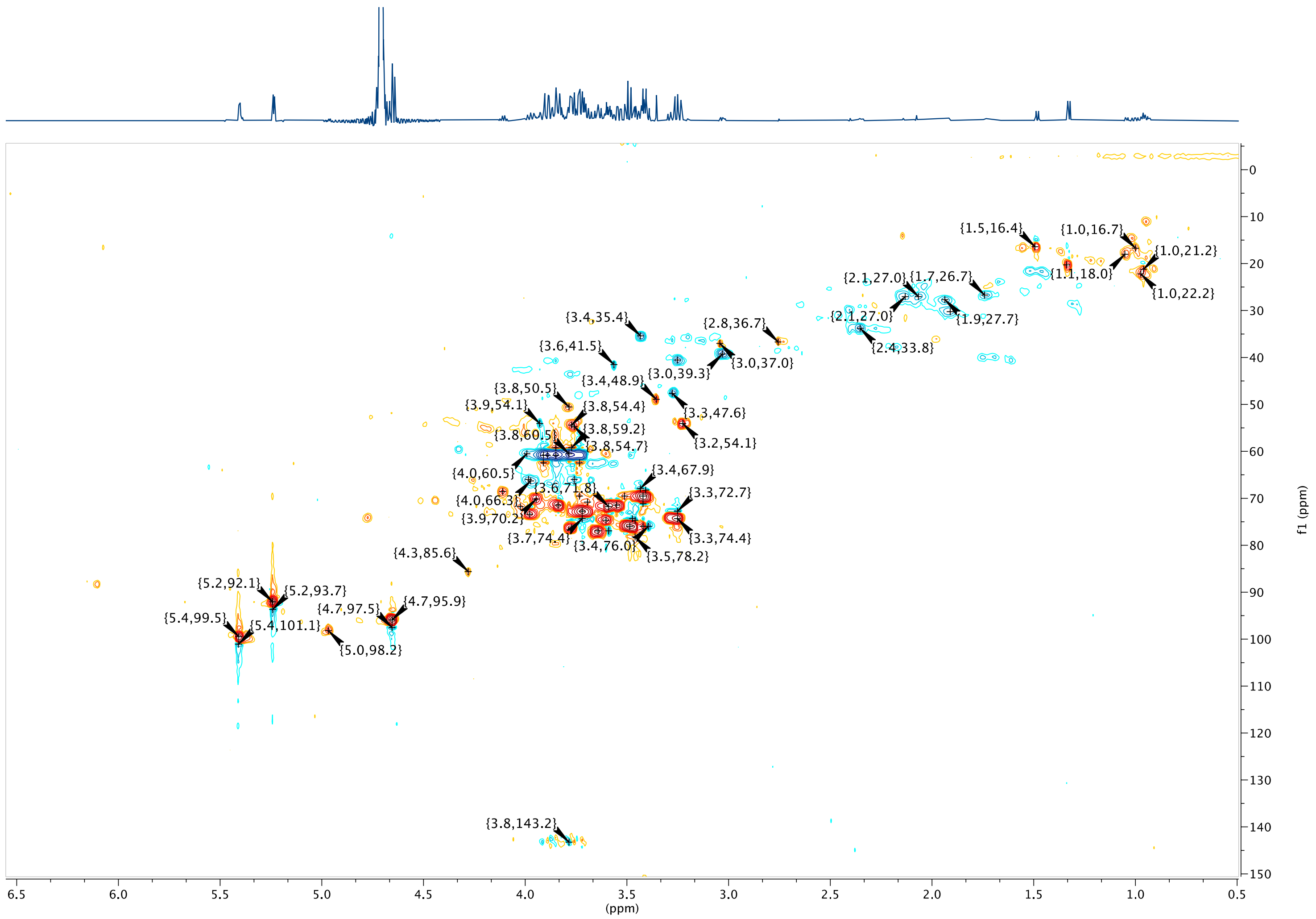

Figure S1. HSQC experiment of control sample recorded at 600 MHz (298 K). ^{1}H and ^{13}C cross peaks (ppm) of main signals are indicated.

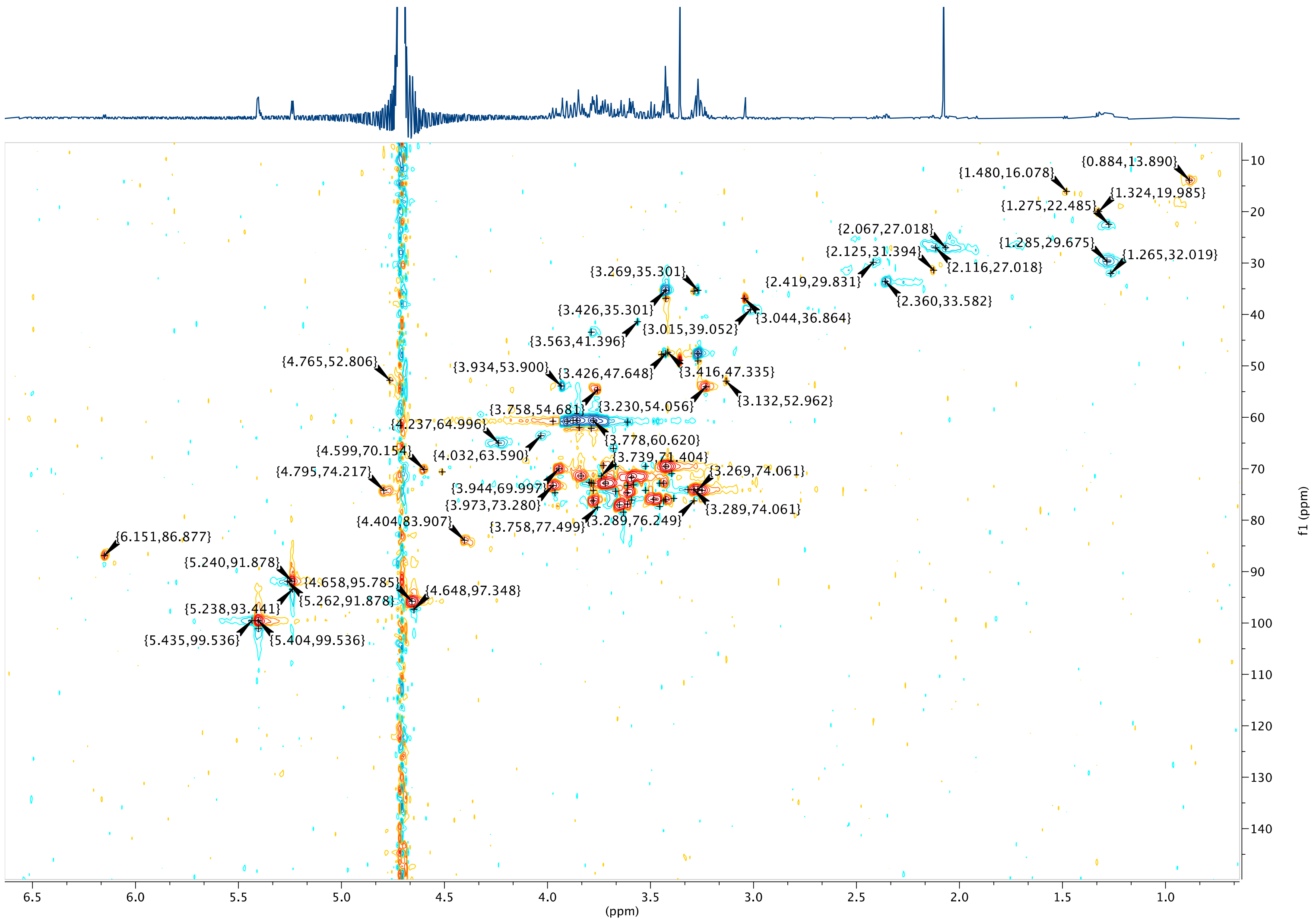

Figure S2. HSQC experiment of toxic sample after 36 days of treatment recorded at 600 MHz (298 K). ^{1}H and ^{13}C cross peaks (ppm) of main signals are indicated.

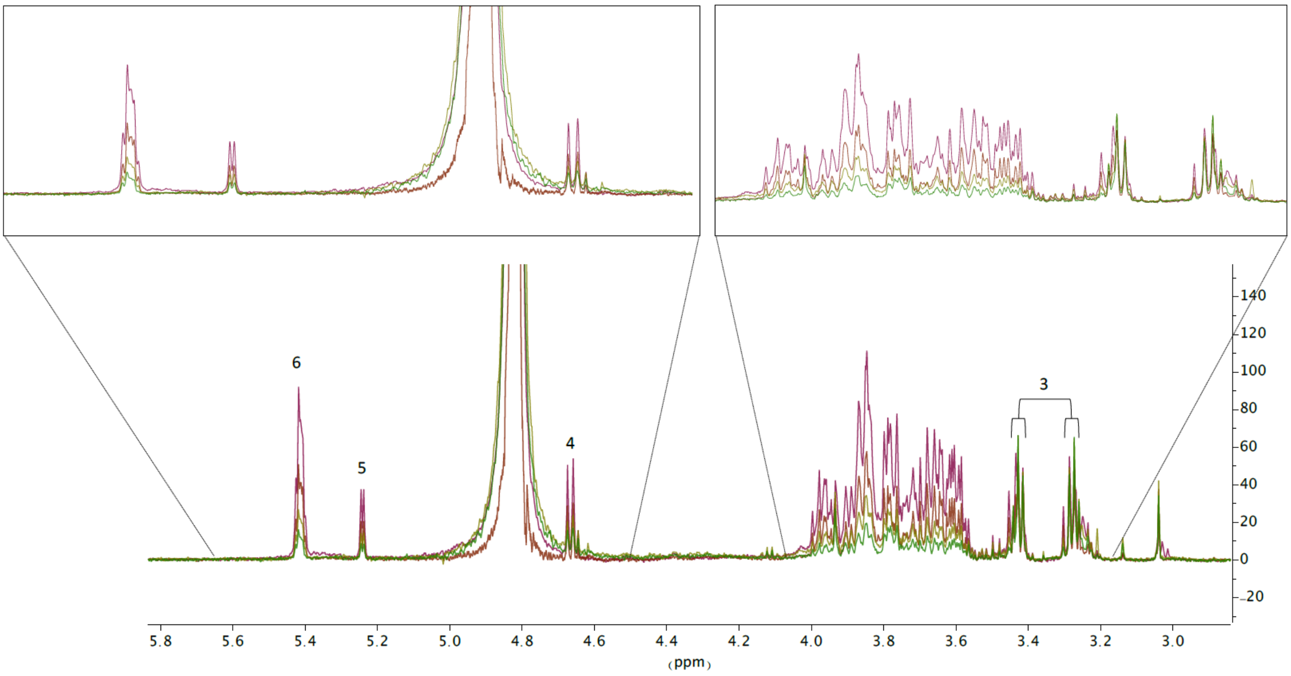

Figure S3. Effect of toxic diet on metabolic profile of liver tissues. Representative normalized ^{1}H NMR spectra (500 MHz, 298 K) of the liver samples of *Carassius auratus* fed with CTX-toxic fish diet. Effect of time of treatment after 8 (green), 15 (ochre), 29 (orange) and 36 (purple) days. Key metabolites: (3) Taurine. (4) α-Glucose. (5) β-Glucose. (6) Glycogen.

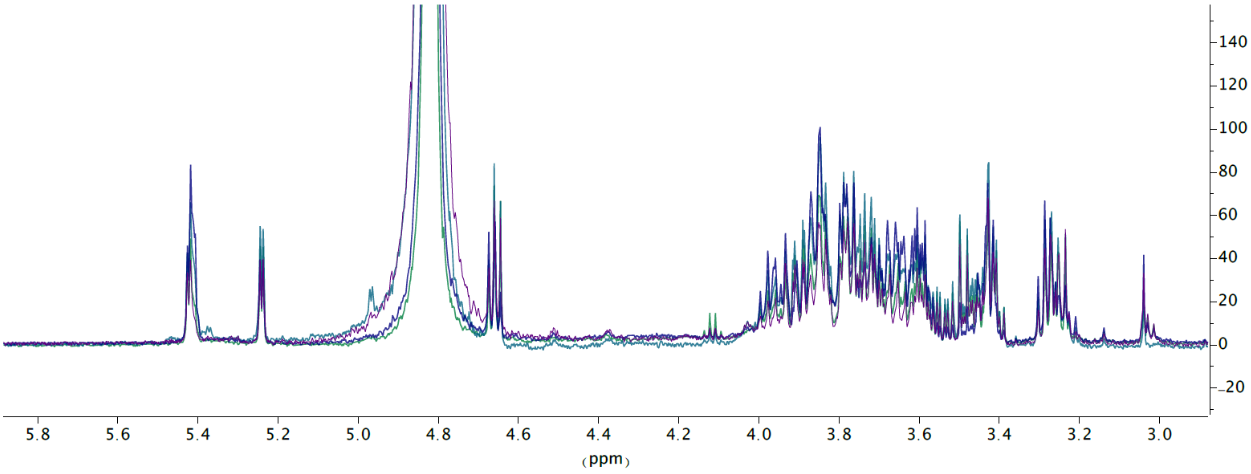

Figure S4. Effect of control diet on metabolic profile of liver tissues. Representative normalized $_{1}$H NMR spectra (500 MHz, 298 K) of the liver samples of *Carassius auratus* fed with non-toxic fish diet. Effect of time after 8 (purple), 15 (blue), 29 (cyan) and 36 (green) days.

Table S2. Ammonia (NH_3) values (ppm) detected in the aquaria with a precision of ± 0.05 ppm at 25 °C. Measurements were done 24 h after the last ingestion, except for the days 0, 6 and 13. Fish were not fed on days 5 and 12 of the experiment. A change of water was performed on day 9.

Sampling day	NH_3 value Toxic aquarium (ppm)	NH_3 value Non-toxic aquarium (ppm)	Differences
0	0.000	0.219	-0.219
1	0.364	0.874	-0.510
2	0.886	1.323	-0.437
3	1.566	1.396	0.170
4	2.197	1.044	1.153
6	3.035	0.219	2.816
7	3.545	0.473	3.072
8	4.176	0.959	3.217
9	4.832	1.433	3.399
9*	1.669	0.983	0.686
10	1.305	0.680	0.625
11	0.388	0.042	0.346
13	0.583	0.000	0.583
14	1.202	0.340	0.862
15	1.785	0.728	1.057

Blue line indicates a water renewal; * the ammonia was measured just after the water change.

Table S3. Water parameters (ammonia (NH_3) values (ppm), oxygen level (ppm), dissolved oxygen saturation (%), temperature (°C), nitrite (NO_2), nitrate (NO_3) (ppm), and pH) detected in the CTX-feed aquaria. Measurements were done 24 h after the last ingestion, except for the days 0, 6 and 13. Fish were not fed on days 5 and 12 of the experiment. A change of water was performed on day 9.

Sampling day	NH_3 value (ppm)	O_2 level (ppm)	Dissolved O_2 saturation (%)	Temperature (° C)	NO_2 (ppm)	NO_3 (ppm)	pH
0	0.000	7.2	85	23.5	0.05	3	6.38
1	0.364						
2	0.886						
3	1.566						
4	2.197	7.2	85	23.8			
6	3.035						
7	3.545						
8	4.176	7.1	84	23.8	0.05	2.5	5.12
9	4.832						
9*	1.669						
10	1.305						
11	0.388	7.3	86	23.4			
13	0.583						
14	1.202						
15	1.785						

Blue line indicates a water renewal; * the ammonia was measured just after the water change.

Table S4. Water parameters (ammonia (NH_3) values (ppm), oxygen level (ppm), dissolved oxygen saturation (%), temperature (°C), nitrite (NO_2), nitrate (NO_3) (ppm), and pH) detected in the non-toxic-feed aquaria. Measurements were done 24 h after the last ingestion, except for the days 0, 6 and 13. Fish were not fed on days 5 and 12 of the experiment. A change of water was performed on day 9.

Sampling day	NH_3 value (ppm)	O_2 level (ppm)	Dissolved O_2 saturation (%)	Temperature (ºC)	NO_2 (ppm)	NO_3 (ppm)	pH
0	0.219	7.1	84	23.8	<0.05	1	5.41
1	0.874						
2	1.323						
3	1.396						
4	1.044	7.1	84	23.9			
6	0.219						
7	0.473						
8	0.959	7.1	85	24.0	0.00	2.5	5.16
9	1.433						
9*	0.983						
10	0.680						
11	0.042	7.3	86	23.7			
13	0.000						
14	0.340						
15	0.728						

Blue line indicates a water renewal; * the ammonia was measured just after the water change.
